# P-980. Film-Based Infectious Diseases(ID) Elective Course

**DOI:** 10.1093/ofid/ofae631.1170

**Published:** 2025-01-29

**Authors:** Su Lee, William Ofstad, Maria Cecilia Barrameda

**Affiliations:** West Coast University School of Pharmacy, Los Angeles, California; West Coast University School of Pharmacy, Los Angeles, California; West Coast University School of Pharmacy, Los Angeles, California

## Abstract

**Background:**

One of innovative educational approaches in enhancing learning outcomes is the integration of cinematic media into educational curricula, to increase student engagement, and motivate students to understand realistic settings to apply their learning (Stone 2010, Karanfil 2016). This research aims to measure qualitative and quantitative assessment of students’ performances in an elective course using movies to introduce various ID in a pharmacy curriculum.

**Methods:**

This study is a systematic analysis of students’ experiences in “PHAR 949 ID in Movies” through assignments and post-course surveys over two years (2023-2024). Students reflected on the course via a post-survey via the CANVAS platform, soliciting feedback on the course experience, including enjoyment, potential areas for improvement, and their knowledge and skills across various ID topics. Students were also asked to indicate how the course had influenced their mindset and future application of learning. Student data were de-identified and thematically analyzed, categorized according to four levels of Kirkpatrick Learning Outcomes.  Quantitative performance assessment for learning was measured with assignments on regarding etiology, transmission, clinical presentation, diagnosis, treatment options, and prophylaxis strategies of each infectious disease topic. All personally identifiable information was removed to ensure confidentiality. IRB#: 05022024_lee_tIDwm.

**Results:**

Ninety-six percent of students were actively engaged in discussions on the films and infectious diseases. Qualitative assessment of post-course survey showed that students had positive responses on Reaction and Learning by 100%, 80% for Behavior, and only 10 % for Results of Kirpatrick Learning Outcomes. In the quantitative assessment, students have achieved the highest average score, 100%, in Cholera (The Painted Veil), followed by COVID-19 (Contagion), 93%, HIV (Philadelphia), 91%, Tuberculosis (Moulin Rouge), 90%, and Syphilis (The English), 89%.

**Conclusion:**

The use of movies to introduce ID in an elective course of a doctoral pharmacy program shows high levels of students’ engagement, reaction, and learning. Students achieved the highest average scores in Cholera (The Painted Veil), followed by COVID-19 (Contagion)

**Disclosures:**

**All Authors**: No reported disclosures

